# Warning Before a Fight: The Role of Distance and Ritualized Agonistic Behaviors in Minimizing Aggression in the Jamaican Fruit Bat

**DOI:** 10.3390/biology14101449

**Published:** 2025-10-20

**Authors:** Orlando R. Vivanco-Montané, Jorge E. Morales-Mávil, Laura T. Hernández-Salazar, Jairo Pérez-Torres, Edgar Ahmed Bello-Sánchez

**Affiliations:** 1 Laboratorio de Biología de la Conducta, Instituto de Neuroetología, Universidad Veracruzana, Avenida Dr. Luis Castelazo Ayala s/n, Colonia Industrial Ánimas, Xalapa 91190, Veracruz, Mexico; orlandovivanco667@gmail.com (O.R.V.-M.); herlatss@gmail.com (L.T.H.-S.); 2 Laboratorio de Ecología Funcional, Unidad de Ecología y Sistemática (UNESIS), Departamento de Biología, Facultad de Ciencias, Pontificia Universidad Javeriana, Carrera 7a No. 43-82, Bogotá 111711, Colombia; jaiperez@javeriana.edu.co; 3 Facultad de Biología-Xalapa, Universidad Veracruzana, Circuito Gonzalo-Aguirre Beltrán S/N, Zona Universitaria, Xalapa 91090, Veracruz, Mexico

**Keywords:** confrontations, harems, dominant males, satellite males, ritualization

## Abstract

In many vertebrates, ritualized behaviors serve to settle conflicts while minimizing the risk of injury. The Jamaican fruit bat (*Artibeus jamaicensis*) is a polygynous species that roosts in caves, where dominant males form and defend harems by displaying agonistic behaviors against satellite males attempting to mate with females. We examined how the distance of satellite males from the harem and the number of females influenced the latency of approach by dominant males during agonistic encounters, and whether these encounters follow a defined behavioral sequence. We analyzed 50 agonistic interactions from video recordings of *A. jamaicensis* harems collected between May and October 2021 in “Cantil Blanco” cave, Veracruz, Mexico. We quantified the number of females per harem and measured the distance of satellite males to the nearest female just before the dominant male initiated an approach. Our results show that satellite male distance determined dominant male approach latency, reflecting a minimum tolerable distance, whereas harem size had no effect. Furthermore, the succession of behaviors observed indicates that these encounters are sequential, escalating from ritualized displays to physical aggression.

## Simple Summary

Animals often compete for mates, and these contests can turn violent. Fighting, however, is risky and can cause serious injuries. To avoid this, many animals use signals and displays to warn rivals before attacking. We studied this process in the Jamaican fruit bat (*Artibeus jamaicensis*), a species where one male defends a group of females inside caves. By watching videos of these bats, we found that the distance of rival males matters; when they get too close, the defending male reacts quickly to chase them away. The number of females in the group did not change the males’ response. These encounters usually follow a precise sequence, beginning with warnings and sometimes ending in fights. Our study reveals how bats manage conflict and reduce the risk of injury.

## 1. Introduction

Agonistic behavior is a social mechanism that regulates group dynamics, arising in competitive contexts and encompassing aggression, submission, defense, and avoidance during confrontations among conspecifics. Such behaviors occur in diverse social situations, including hierarchy formation, territorial defense and competition for females [1,2,3].

In most mammals, the number of matings determines the reproductive success in males [4]. In gregarious species, this often leads to intense competition for access to females [4,5,6]. Because confrontations are energetically costly and involve the risk of injury, contestants frequently rely on ritualized behaviors to resolve conflicts without escalating to physical fights [7,8]. Ritualization is an evolutionary process in which behavioral patterns are modified to enhance communication [9,10]. These ritualized encounters consist of successive stages that reveal contestants’ motivation and resource-holding potential, or fighting ability [8], allowing each participant to reassess at each stage whether to persist or withdraw.

Among bats, various mating systems have been described [11], with polygyny being the most common [12,13]. In this system, a single male mates with multiple females [14,15] and aggressive encounters among adult males are particularly frequent during the reproductive season [11,16,17,18,19,20]. The Jamaican fruit bat (*Artibeus jamaicensis*) is a polygynous species that forms cave-dwelling harems composed of a dominant male defending 4 to 18 females. In larger groups (>14 females), a subordinate male is often present, typically smaller, and gains reproductive opportunities with some females through association with the dominant male [17]. Dominant and subordinate males remain closely associated with their harems, displaying agonistic behaviors toward satellite males, which approach different harems in search of mating opportunities [18]. By focusing their defense on a fixed group of females, they do not compete with others for every copulation. Consequently, encounters with satellite males are likely ritualized, involving warning postures and displays that prevent escalation to costly physical aggression [8].

Here, we investigate the relationship between satellite male distance from the harem and the number of females with the latency of approach by the dominant male during agonistic encounters, as well as the sequence of behaviors exchanged between dominant and satellite males.

## 2. Materials and Methods

### 2.1. Study Site

**Figure 1:**
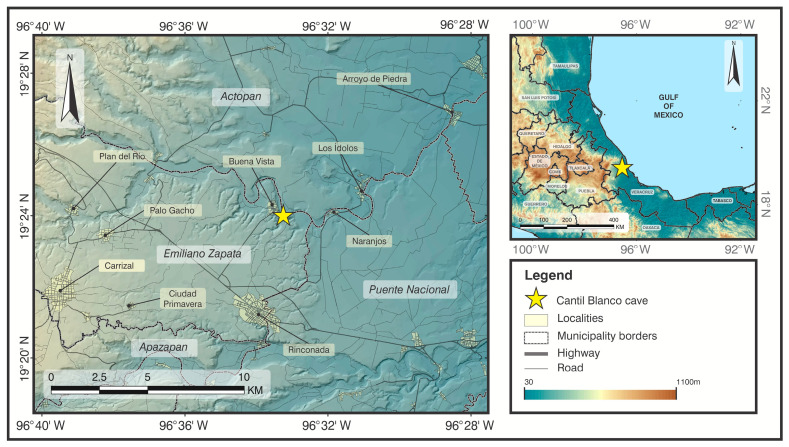
Location of the Cantil Blanco cave at Buena Vista, Emiliano Zapata.

Fieldwork was conducted between May and October 2021, coinciding with the peak reproductive activity of *Artibeus jamaicensis* [17,21,22], in Cantil Blanco cave, located in Buena Vista, Emiliano Zapata, Veracruz, Mexico (19°23′59″ N, 96°33′15.31″ W; WGS84, Figure 1). This cave extends to a depth of 12 m, starting from an entrance approximately 3 m high and 3 m wide, narrowing to 1 m high and 1.5 m wide at its most distal point. Situated along the Paso de la Milpa River within a fragment of tropical dry forest. The surrounding landscape is dominated by agriculture (55%), followed by grasslands (26%), tropical dry forest (9%), oak forest (7%), and urban areas (2%) [23]. The cave supports a large population of *A. jamaicensis*, composed of harems as well as diffuse groups of juveniles, females, and bachelor males. Harems are distributed throughout the cave, most commonly occupying small cavities in the ceiling. Other bat species, including *Pteronotus personatus* and *Desmodus rotundus*, also roost in the cave.

### 2.2. Behavioral Data Collection

We carried out behavioral observations every 15 days. Video recordings lasting 10–15 min were made of harems present during sampling sessions using a digital camera (Andoer HDV-301LTRM, Shenzhen, China) equipped with additional infrared illumination (Ordro Ln-3, Ordro, Shenzhen, China), recording at 1080 p and 30 fps, mounted on a tripod [24]. To minimize disturbance ensuring comprehensive recording of agonistic interactions, we entered the cave exclusively for camera repositioning. We analyzed the recordings with UVehavior software, Version 1.0.0 [25]. Focal sampling was applied to adult males, while all-occurrence sampling was used to record agonistic behaviors [26]. From the videos, we quantified the number of females in each harem. We measured the distance between satellite males and the nearest harem member immediately prior to the dominant male’s approach, using the open-source software ImageJ 1.53e [27,28]. For each interaction, we selected the video frame immediately prior to the dominant male’s approach to the satellite. The linear distance between the midpoint of the thorax of the two individuals was measured in pixels. To calibrate these measurements, we used the forearm length of individuals visible in the recordings as a reference. The pixel length of a forearm was measured in ImageJ and scaled using the mean forearm length (mean ± SD = 60.6 ± 2.29) of adult males previously captured for other studies in the same population.

### 2.3. Behavioral Descriptions and Variables Recorded

We described the agonistic encounters based on observed behaviors, supplemented with behaviors previously reported for *A. jamaicensis*—such as wing-flicks, chasing, and confrontation [18]—as well as the repertoire described for *Carollia perspicillata* [20]. For each encounter, we recorded the frequency of behaviors and the latency of approach by the dominant male after detecting the presence of a satellite male. We also documented transitions between successive behaviors to assess whether encounters followed a consistent sequence indicative of ritualized patterns [20].

### 2.4. Statistical Analysis

To examine the relationship between satellite male distance from the harem and the latency of approach by the dominant male, as well as between harem size and approach latency, we conducted a model comparison analysis. After evaluating multiple modeling approaches, including linear, quadratic, and other non-linear specifications, we found that quadratic regression provided the best fit. To test whether behavioral sequences followed consistent patterns, we estimated transition probabilities among stages using Markov chains [29] and compared observed first-order transitions with random expectations using Chi-square tests [20]. All analyses were conducted with a 95% confidence level in R version 4.2.2 via the RStudio Version 1.1.442 interface [30,31], employing the markovchain package and ggplot2 for data visualization [32,33].

## 3. Results

We obtained a total of 127 videos, corresponding to approximately 1500 min of recordings. Of these, 29 videos contained agonistic interactions between males with sufficient quality for detailed analysis.

### 3.1. Behavioral Repertoire

**Figure 2:**
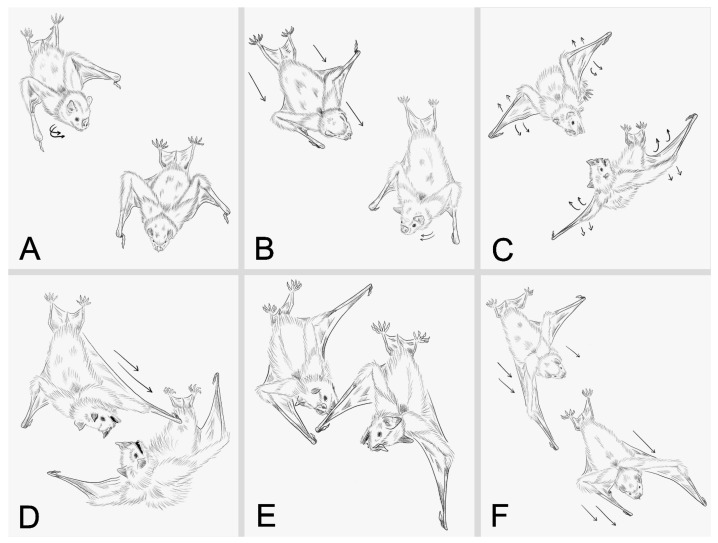
Illustrated behavioral repertoire of agonistic interactions in male *Artibeus jamaicensis*. (**A**) Detection, (**B**) Approach, (**C**) Wing-flicks, (**D**) Boxing, (**E**) Biting, and (**F**) Chasing. Illustrations were based on still frames extracted from the video recordings used for behavioral analysis. The arrows show the direction and movement of the bats.

A total of 50 agonistic interactions of variable duration were analyzed (mean ± SD = 27.54 ± 24.05 s), all of which were observed within the 29 videos. These interactions were composed of six distinct behaviors (Table 1; Figure 2; Video S1). We found significant differences in the frequencies of behaviors displayed during agonistic interactions (χ^2^ = 108.39, *p* < 0.001). The most frequently observed behaviors were wing-flicks and approach, whereas biting and chasing were the least frequent.

### 3.2. Latency to Approach

The distance of the satellite male from the group (mean ± SD = 98.66 ± 55.49 mm) showed a quadratic relationship (Figure S1) with the approach latency of the dominant male (mean ± SD = 4.48 ± 8.43 s) (F = 11.600; df = 2.19; R^2^ = 0.55; *p* < 0.001). Approach latencies rarely exceeded 5 s. Notably, latencies were shortest when satellites were within ~100 mm of the harem, indicating that this distance may represent a critical threshold for eliciting rapid responses by dominant males. Likewise, the analysis showed no relationship between the number of females in the harem (mean ± SD = 16.79 ± 5.97), which varied between 11 and 26 individuals, and the latency to approach (mean ± SD = 2.96 ± 4.69 s) by the dominant male (F = 0.07769; df = 17; R^2^ = 0.054; *p* = 0.784).

### 3.3. Sequence of Behavioral Stages

In 15 of the 50 interactions (30%), encounters did not escalate to physical contact between males. In eight cases (16%), the encounter progressed directly from detection to boxing/biting or began with boxing, without warning behaviors by the dominant male following the satellite male’s approach. Only two encounters (4%) escalated to the final level (chasing). The rest of interactions (50%) began with a warning prior to contact and ended in biting behavior.

**Figure 3:**
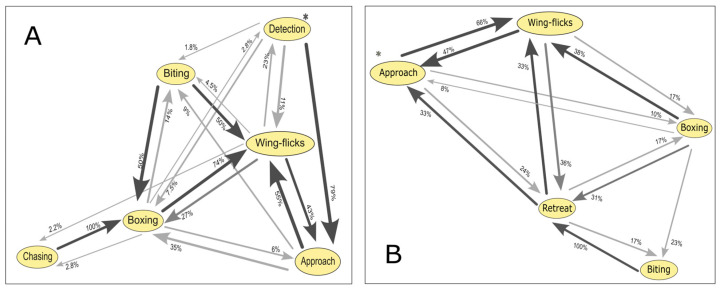
Transition probabilities between behavioral states during agonistic encounters in *Artibeus jamaicensis*. Each node represents a recorded behavior, and arrows indicate the probability of transitioning from one behavior to another; arrow thickness is proportional to transition probability: (**A**) Dominant males. (**B**) Satellite males. Encounters begin at the behavior marked with an asterisk (*).

For dominant males, early stages of interaction showed a higher probability of first-order transitions which represent changes from one behavioral state to another completely different one with no gradual intermediate state that follows a natural escalation of aggression (e.g., detection to approach, 79%; approach to wing-flicks, 43%) compared to random transitions (e.g., detection to wing-flicks, 11%; detection to biting, 1.8%; approach to boxing, 35%) (Figure 3A). For the biting and chasing behaviors, no upward transitions were recorded, but random transitions to lower levels occurred. First-order transitions significantly diverged from random expectations in most cases (Table 2), except for the transition approach to wing-flicks (χ^2^ = 0.55; *p* = 0.45). In contrast, for boxing to biting and biting to chasing, random transitions were more frequent than first-order transitions.

For satellite males, the probability of first-order transitions exceeded random expectations only in approach to wing-flicks (66%) and biting to retreat (100%) (Figure 3B). First-order transitions significantly diverged from random expectations in all cases (Table 3), except for boxing to biting (χ^2^ = 3.769; *p* = 0.52). However, in the cases of approach to wing-flicks and in wing-flicks to boxing, random transitions were more frequent than first-order ones.

## 4. Discussion

The behavioral repertoire of *Artibeus jamaicensis* during agonistic interactions comprises six behaviors, each with varying levels of threat and aggression. The number of behaviors recorded was like those reported for *Carollia perspicillata* [20] and for other populations of *A. jamaicensis* [18]. These behaviors express the intention to initiate or continue a confrontation, as well as fighting ability, through explicit bodily displays and physical contact, with different successions throughout the encounters.

We recorded a negative influence of satellite males on dominant males, where the latter reduced their latency to approach in response to the satellite’s proximity to the group, which suggests the existence of a territorial boundary that triggers defensive responses [34,35]. In birds such as the Carolina wren (*Thryothorus ludovicianus*) or the hooded warbler (*Wilsonia citrina*), simulated intrusions into the territory trigger faster approaches or greater aggression towards neighboring or unfamiliar conspecifics [34,35,36]. Similarly, in mammals such as the desert pocket mouse (*Chaetodipus siccus*) and the banner-tailed kangaroo rat (*Dipodomys spectabilis*), the defense of “core areas” around feeding sites or burrow entrances has been observed [35,37]. In our observations of *A. jamaicensis*, this is evident when intruding bats perch farther from harems: in such cases, dominant males usually remain alert or display warning signals, approaching slowly only if stronger actions are required to deter opponents. However, when intruders perch closer to the group, dominant males must approach more quickly, as this could represent a more serious threat due to the increased probability of the intruder copulating with females from the harem.

The absence of association between harem size and the latency of dominant males in approaching satellites can be attributed to the behavior observed in some polygynous bat species. In these species, males do not prevent females from moving between groups, –which leads to female turnover. This behavior has been documented in the short-tailed fruit bat (*Carollia perspicillata*) and the greater spear-nosed bat (*Phyllostomus hastatus*) [18,38,39]. In *A. jamaicensis*, polygyny in caves has been proposed to involve female defense, since roost availability is not usually a limiting factor for group formation [40]. However, our study cave, “Cantil Blanco”, is small and hosts a high density of individuals, which may lead to intense competition for space and less stable harems compared to other caves [17,18]. Competition for roosting sites inside caves may be influenced by a combination of physical, spatial, and environmental variables, as observed in *C. perspicillata*, which exhibits preferences for cavities with specific features such as rough textures [41]. Thus, it is likely that *A. jamaicensis* males prefer to defend roosting sites containing a harem to which they exhibit high fidelity, regardless of the number of females, if this number fluctuates frequently. For example, in short-tailed fruit bats, females may change harems an average of three to four times over six months, resulting in unstable group membership [11].

Sexual selection processes and social dynamics in bats can be highly variable, even among polygynous species. From the male perspective, both resource-defense and female-defense polygyny have been described [18,20]. In either case, male potential to monopolize females as mating partners depends not only on the outcome of male–male contests but also on constant assessment of male condition by females. For instance, *Noctilio* spp. and *Leptonycteris curasoae* produce odors from inguinal glands or dorsal patches, respectively, to mark their territories and signal ownership of female groups [42,43]. Females are thought to use male scent glands in *Noctilio* spp. to assess mate quality, while in *L. curasoae* females assess males with larger dorsal patches, as patch size may indicate health [44]. In *Artibeus* spp., no distinct traits are currently known to be utilized by females for mate assessment, although body mass or vocal characteristics associated with mass could potentially serve this role [45].

Regarding agonistic interactions between *A. jamaicensis* males, these can be divided into two phases. The first stage consists of a non-contact, composed of “detection”, “approach”, and “wing-flicks”. In this stage, first-order transitions were more frequent than random ones, although there was a high probability of reverse transitions from “wing-flicks” to “approach”. This sequence is typical of ritualized behaviors and is consistent with descriptions of agonistic interactions in mammals such as domestic pigs [10], deer [46,47], and the short-tailed fruit bat [20], where confrontations typically begin without direct physical contact.

The second phase involves contact behaviors, including “boxing”, “biting” and “chasing”. During this stage, the probabilities of first-order transitions are low, indicating a higher likelihood of returning to “wing-flicks” after “boxing” or “biting”, rather than escalating the situation. This observation supports the idea that ritualized displays often become stereotyped and incomplete [48], with individuals repeating certain behaviors instead of utilizing their full range of behavioral options. A similar pattern occurs in the European fallow deer (*Dama dama*), where males may interrupt a fight to return to the ritualized “parallel walk” display, which functions as an exhibition and allows assessment of fighting ability [47].

The “wing-flicks” behavior recorded in this study has also been described in other species such as the broad-eared free-tailed bat (*Nyctinomops laticaudatus*) [19], the short-tailed fruit bat (*Carollia perspicillata*) [20], and the great Himalayan leaf-nosed bat (*Hipposideros armiger*) [48] in similar contexts. In *A. jamaicensis*, detailed observations described stereotyped agonistic responses in which short, rapid wing-flicks were emitted together with vocalizations and brief chases or attempts to bite, especially toward visiting adult males during the breeding season [18]. This behavior involves upright postures that may allow opponents to assess body condition. Continuous updating during interactions, by returning to or repeating wing-flicks, allows each participant to decide whether to withdraw or escalate the situation [8]. Additionally, since vocalizations often accompany wing-flicks and acoustic signals may also be used to assess fighting ability, as they depend on an individual’s condition [49]. In many species, individuals in better health with greater body mass produce lower-frequency vocalizations, acting as honest signals [48,49].

Our findings demonstrate that agonistic interactions in *A. jamaicensis* follow a structured sequence that combines spatial constraints with ritualized behavioral stages. The two-phase organization in the encounters, composed of warning signals and direct physical aggression, resembles patterns described in other mammals with ritualized patterns. Such ritualization, likely reinforced by visual and acoustic signals, serves to mediate conflicts, reduce energetic costs, and minimize injury risk, while maintaining access to reproductive opportunities.

## 5. Conclusions

Our results provide a comprehensive overview of agonistic interactions among A. jamaicensis males during the breeding season. We found that the distance of satellite males from the harem influenced the dominant male’s approach latency and reflects a minimum tolerable distance of around 100 mm. However, harem size had no significant effect on approach latency. Furthermore, encounters followed a defined sequence of stages. The most frequent behavior was wing-flicks, which we interpret to be a behavior that could be used to assess fighting ability and resource-holding potential. This is consistent with descriptions of ritualized agonistic encounters across different taxa. These findings emphasize the role of ritualized agonistic behavior in structuring male–male competition in *A. jamaicensis*. This behavioral organization shapes social interactions and reproductive dynamics, while also mitigating the risk of escalated conflict.

## Figures and Tables

**Table 1:** Behavioral repertoire of *Artibeus jamaicensis* during harem-defense agonistic interactions.

Behavior	Description
Detection	A satellite male is approaching the group. The dominant male turns his ears and head towards the opponent’s position. At this stage, the competitors are usually separated by more than one wingspan, so physical contact is impossible.
Approach	When the opponent moves towards the group, the dominant male moves closer to reduce the distance between them.
Wing-flicks	With their wings partially extended, the opponents flap rapidly in a manner resembling strikes. The chest and neck are extended towards the opponent between the wings, often accompanied by vocalizations. Although no physical contact occurs, wing-flicks may alternate with occasional boxing and vocalizations.
Boxing	The conflict escalates to involve physical contact through wrist strikes. Both wings are partially extended with the chest protruding forward. One forearm delivers rapid, repeated blows towards the opponent while the other remains partially extended, either flapping quickly or holding onto the cave wall. This behavior is frequently accompanied by vocalizations.
Biting	If the intruder persists in approaching the group, the dominant male, with his wings partially extended, attempts to bite the opponent’s neck or forearms. These biting attempts are often interspersed with directed wing flaps.
Chasing	When one opponent retreats, the other pursues them, often continuing the wing flaps until they have gone completely.
Retreat	When one opponent moves away from the other individual in conflict.

**Table 2:** First-order vs. random transitions by dominant males.

Transition	First-Order	Random	χ^2^	*p*
Detection-Approach	42	11	18.132	<0.001
Approach-Wing-flicks	25	20	0.555	0.456
Wing-flicks-Boxing	12	32	9.091	0.002
Boxing-Biting	5	30	17.857	<0.001
Biting-Chasing	0	6	6	0.014

**Table 3:** First-order vs. random transitions by satellite males.

Transition	First-Order	Random	χ^2^	*p*
Detection-Approach	27	14	4.022	0.042
Approach-Wing-flicks	6	30	16	<0.001
Wing-flicks-Boxing	3	10	3.769	0.052
Boxing-Biting	5	0	5	0.025

## Data Availability

The data included in this study are the property of the universities UV and PUJ and can be obtained by contacting the corresponding author at jormorales@uv.mx or ebello@uv.mx upon request.

## Supplementary Materials

The following supporting information can be downloaded at: https://www.mdpi.com/article/10.3390/biology14101449/s1. Video S1. Agonistic interaction between a dominant and satellite male in *A. jamaicensis.* Can be downloaded at https://doi.org/10.5281/zenodo.17354066. Figure S1. Quadratic regression plot showing the relationship between dominant male’s latency to approach and distance of satellite male. Can be downloaded at https://doi.org/10.5281/zenodo.17354920.
