# Supplementary material for: Warning Before a Fight: The Role of Distance and Ritualized Agonistic Behaviors in Minimizing Aggression in the Jamaican Fruit Bat
Source: Biology (Basel). 2025 Oct 20;14(10):1449. doi: 10.3390/biology14101449 (PMC12561160; doi:10.3390/biology14101449)
Supplement: Supplementary file 1 [file biology-14-01449-s001.zip › biology-3918288-supplementary.pdf]

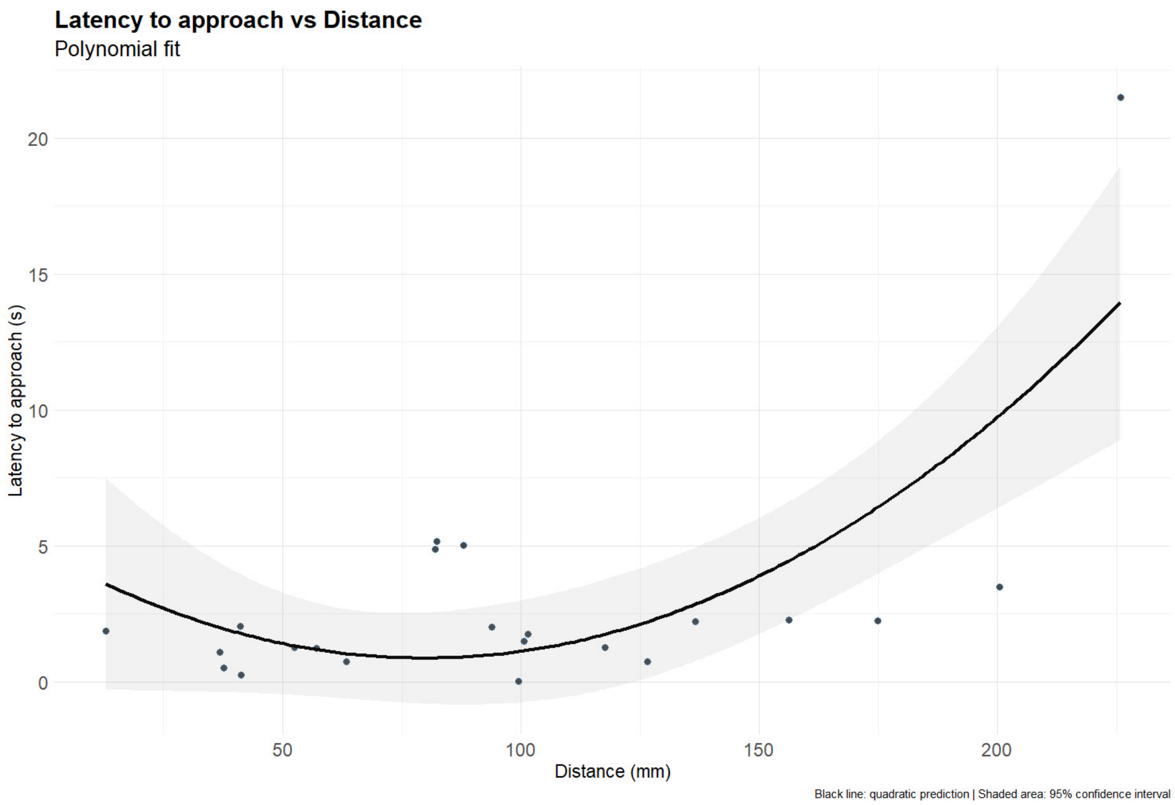

Figure S1. The distance of the satellite male from the group (mean ± SD: 98.66 ± 55.49 mm) exhibited a quadratic relationship with the approach latency of the dominant male (mean ± SD: 4.48 ± 8.43 s) ($F = 11.60$; $df = 2.19$; $R^2 = 0.55$; $p < 0.001$).

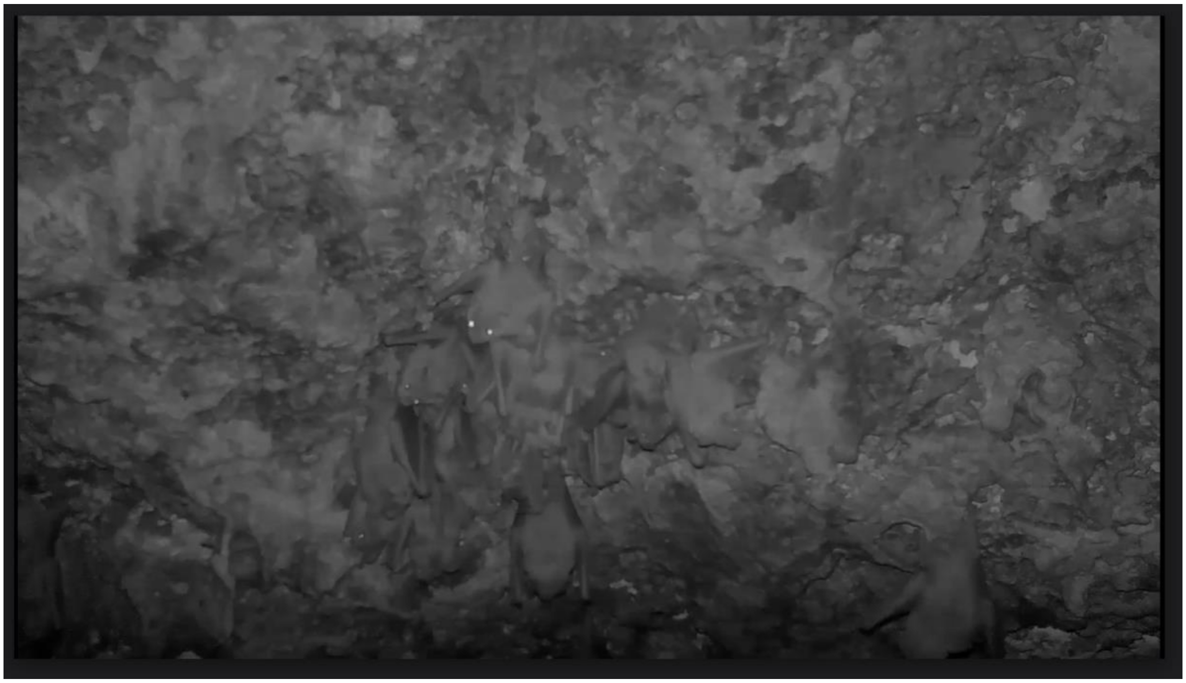

Video S1. Agonistic interaction between a dominant and satellite male in *A. jamaicensis*

https://doi.org/10.5281/zenodo.17354066
